# Association of Blood Biomarkers With Acute Sport-Related Concussion in Collegiate Athletes: Findings From the NCAA and Department of Defense CARE Consortium

**DOI:** 10.1001/jamanetworkopen.2019.19771

**Published:** 2020-01-24

**Authors:** Michael McCrea, Steven P. Broglio, Thomas W. McAllister, Jessica Gill, Christopher C. Giza, Daniel L. Huber, Jaroslaw Harezlak, Kenneth L. Cameron, Megan N. Houston, Gerald McGinty, Jonathan C. Jackson, Kevin Guskiewicz, Jason Mihalik, M. Alison Brooks, Stephan Duma, Steven Rowson, Lindsay D. Nelson, Paul Pasquina, Timothy B. Meier, and the CARE Consortium Investigators, Tatiana Foroud, Barry P. Katz, Andrew J. Saykin, Darren E. Campbell, Steven J. Svoboda, Joshua Goldman, Jon DiFiori

**Affiliations:** 1 Department of Neurosurgery, Medical College of Wisconsin, Milwaukee; 2 Michigan Concussion Center, University of Michigan, Ann Arbor; 3 Department of Psychiatry, Indiana University School of Medicine, Indianapolis; 4 National Institute of Nursing Research, National Institutes of Health, Bethesda, Maryland; 5 UCLA Steve Tisch BrainSPORT Program, Departments of Neurosurgery and Pediatrics, University of California, Los Angeles; 6 Department of Epidemiology and Biostatistics, School of Public Health–Bloomington, Indiana University, Bloomington; 7 Keller Army Community Hospital, West Point, New York; 8 Air Force Academy, Colorado Springs, Colorado; 9 Matthew Gfeller Sport-Related Traumatic Brain Injury Research Center, Department of Exercise and Sport Science, University of North Carolina at Chapel Hill, Chapel Hill; 10 Department of Orthopedics and Rehabilitation, School of Medicine and Public Health, University of Wisconsin, Madison; 11 Department of Biomedical Engineering, Virginia Polytechnic Institute and State University, Blacksburg; 12 Department of Physical Medicine and Rehabilitation, Uniformed Services University, Bethesda, Maryland

## Abstract

**Importance:**

There is potential scientific and clinical value in validation of objective biomarkers for sport-related concussion (SRC).

**Objective:**

To investigate the association of acute-phase blood biomarker levels with SRC in collegiate athletes.

**Design, Setting, and Participants:**

This multicenter, prospective, case-control study was conducted by the National Collegiate Athletic Association (NCAA) and the US Department of Defense Concussion Assessment, Research, and Education (CARE) Consortium from February 20, 2015, to May 31, 2018, at 6 CARE Advanced Research Core sites. A total of 504 collegiate athletes with concussion, contact sport control athletes, and non–contact sport control athletes completed clinical testing and blood collection at preseason baseline, the acute postinjury period, 24 to 48 hours after injury, the point of reporting being asymptomatic, and 7 days after return to play. Data analysis was conducted from March 1 to November 30, 2019.

**Main Outcomes and Measures:**

Glial fibrillary acidic protein (GFAP), ubiquitin C-terminal hydrolase-L1 (UCH-L1), neurofilament light chain, and tau were quantified using the Quanterix Simoa multiplex assay. Clinical outcome measures included the Sport Concussion Assessment Tool–Third Edition (SCAT-3) symptom evaluation, Standardized Assessment of Concussion, Balance Error Scoring System, and Brief Symptom Inventory 18.

**Results:**

A total of 264 athletes with concussion (mean [SD] age, 19.08 [1.24] years; 211 [79.9%] male), 138 contact sport controls (mean [SD] age, 19.03 [1.27] years; 107 [77.5%] male), and 102 non–contact sport controls (mean [SD] age, 19.39 [1.25] years; 82 [80.4%] male) were included in the study. Athletes with concussion had significant elevation in GFAP (mean difference, 0.430 pg/mL; 95% CI, 0.339-0.521 pg/mL; *P* < .001), UCH-L1 (mean difference, 0.449 pg/mL; 95% CI, 0.167-0.732 pg/mL; *P* < .001), and tau levels (mean difference, 0.221 pg/mL; 95% CI, 0.046-0.396 pg/mL; *P* = .004) at the acute postinjury time point compared with preseason baseline. Longitudinally, a significant interaction (group × visit) was found for GFAP (*F*_7,1507.36_ = 16.18, *P* < .001), UCH-L1 (*F*_7,1153.09_ = 5.71, *P* < .001), and tau (*F*_7,1480.55_ = 6.81, *P* < .001); the interaction for neurofilament light chain was not significant (*F*_7,1506.90_ = 1.33, *P* = .23). The area under the curve for the combination of GFAP and UCH-L1 in differentiating athletes with concussion from contact sport controls at the acute postinjury period was 0.71 (95% CI, 0.64-0.78; *P* < .001); the acute postinjury area under the curve for all 4 biomarkers combined was 0.72 (95% CI, 0.65-0.79; *P* < .001). Beyond SCAT-3 symptom score, GFAP at the acute postinjury time point was associated with the classification of athletes with concussion from contact controls (β = 12.298; 95% CI, 2.776-54.481; *P* = .001) and non–contact sport controls (β = 5.438; 95% CI, 1.676-17.645; *P* = .005). Athletes with concussion with loss of consciousness or posttraumatic amnesia had significantly higher levels of GFAP than athletes with concussion with neither loss of consciousness nor posttraumatic amnesia at the acute postinjury time point (mean difference, 0.583 pg/mL; 95% CI, 0.369-0.797 pg/mL; *P* < .001).

**Conclusions and Relevance:**

The results suggest that blood biomarkers can be used as research tools to inform the underlying pathophysiological mechanism of concussion and provide additional support for future studies to optimize and validate biomarkers for potential clinical use in SRC.

## Key Points

**Question:**

Is sport-related concussion associated with levels of traumatic brain injury biomarkers in collegiate athletes?

**Findings:**

In this case-control study of 504 collegiate athletes with concussion, contact sport control athletes, and non–contact sport athletes, the athletes with concussion had significant elevations in multiple traumatic brain injury biomarkers compared with preseason baseline and with 2 groups of control athletes without concussion during the acute postinjury period.

**Meaning:**

These results suggest that blood biomarkers can be used as research tools to inform the underlying pathophysiological mechanism of concussion and provide additional support for future studies to optimize and validate biomarkers for potential clinical use in sport-related concussion.

## Teaser

This case-control study examines the association between sport-related concussion and levels of traumatic brain injury biomarkers in collegiate athletes.

## Introduction

During the past decade, several candidate biomarkers have emerged as potential diagnostic markers of traumatic brain injury (TBI).^1,2,3,4^ Biomarkers have provided insight into the underlying pathophysiological mechanisms of TBI, particularly the mechanisms and dynamic course of neuronal, axonal, and astroglial damage that result from TBI.^5^

An estimated 90% of brain injuries are classified as mild TBI. Early work has identified select biomarkers with favorable performance in the context of mild TBI, including glial fibrillary acidic protein (GFAP), ubiquitin C-terminal hydrolase-L1 (UCH-L1), neurofilament light chain (NF-L), and S100B.^5,6,7^ Previous studies^8,9,10^ have reported high sensitivity and negative predictive value of UCH-L1, GFAP, NF-L, and tau biomarkers to predict intracranial injury present on acute-phase head computed tomography.

Sport-related concussion (SRC) represents a sizeable subset of mild TBI, affecting millions of athletes per year. Within the spectrum of TBI, SRC reflects the most subtle and mild form of mild TBI; consequently, diagnosing SRC is often challenging because of the need to rely on subjective self-reported symptoms that are not specific to mild TBI and the limited diagnostic utility of common clinical tests.^11^ As in the broader mild TBI spectrum, diagnostic biomarkers have potential to objectively assess SRC presence and severity and to monitor recovery while also advancing our understanding of the underlying pathophysiological mechanisms of concussion. A previous systematic review^12^ found several studies that reported alterations in various biomarkers during the acute phase after SRC, but the strength and generalizability of their findings are limited by small sample sizes, varied methods, and other factors. That review^12^ concluded that there was early, but still limited, evidence that biomarkers have significant promise as research tools for studying SRC but require larger-scale validation to determine their ultimate clinical utility.

The National Collegiate Athletic Association (NCAA) and US Department of Defense (DoD) Concussion Assessment, Research, and Education (CARE) Consortium is a large-scale investigation of the acute effects of SRC in NCAA student athletes and military service academy cadets.^13^ The current study investigated the association of acute SRC with blood biomarkers in collegiate varsity athletes compared with 2 closely matched athlete control groups without SRC. We hypothesized that targeted biomarkers would have elevated serum levels in athletes with concussion during the acute phase compared with preseason baseline levels and with levels in control athletes without concussion.

## Methods

This multicenter, prospective, case-control study was conducted by the NCAA-DoD CARE Consortium from February 20, 2015, to May 31, 2018, at 6 CARE Advanced Research Core (ARC) sites.^13^ This study was approved by the Medical College of Wisconsin institutional review board and the human research protection office. Written informed consent was obtained from all participants. All data were deidentified. Data analysis was conducted from March 1 to November 30, 2019. This study followed the Strengthening the Reporting of Observational Studies in Epidemiology (STROBE) reporting guideline.^14^

### Participants

A total of 1760 athletes completed baseline clinical assessments and blood sample collection. Of these, 504 athletes with concussion, contact sport control athletes, and non–contact sport control athletes also completed clinical testing and blood sample collection at the acute postinjury period, 24 to 48 hours after injury, the point of reporting being asymptomatic, and 7 days after return to play. Concussion was defined according to the consensus definition from the DoD evidence-based guidelines.^15^ Both contact sport controls and non–contact sport controls were matched to athletes with concussion by institution, sex, race/ethnicity, and baseline score on the Wechsler Adult Reading Test.^16^ In addition, contact sport controls were matched to athletes with concussion based on sport, position, years of participation, and concussion history. Non–contact sport controls were recruited from sports with similar exertional requirements but without head impact exposure, including baseball or softball, basketball, and track and field. Both control groups underwent the same clinical protocol for baseline and serial assessment. Only athletes with concussion and contact sport control athletes had blood samples collected at baseline.

### Procedures

The CARE ARC protocol involves preseason baseline clinical testing and blood biospecimen collection in contact sport athletes from football, soccer, lacrosse, ice hockey, rugby, and wrestling. The CARE ARC postinjury protocol includes follow-up clinical testing and blood collection in athletes with concussion at several time points: (1) acute postinjury time point, (2) 24 to 48 hours after injury, (3) the point of reporting being asymptomatic and the start of the return-to-play (RTP) protocol, (4) 7 days after unrestricted RTP, and (5) 6 months after injury. Not all participants completed all visits. Sample sizes for blood sample collection at each visit are presented in eTable 4 in the Supplement.

### Clinical Data Collection

Athletes with concussion and controls were administered the Sport Concussion Assessment Tool–Third Edition (SCAT-3) symptom evaluation, the Standardized Assessment of Concussion (SAC), and the Balance Error Scoring System (BESS) at all time points. The Brief Symptom Inventory 18 (BSI-18) was administered at all time points except the acute postinjury visit.

### Blood Sample Collection

Nonfasting blood samples were collected by venipuncture at baseline and all postinjury time points. A 10-mL red-top tube for serum was collected at each time point, centrifuged within 60 minutes of collection for 15 minutes at 1500*g*, and then aliquoted into cryovials. The cryovials were stored upright in a −27 °C freezer until shipped on dry ice to the CARE Consortium biorepository at Indiana University School of Medicine for long-term storage and remained frozen until analysis.

### Biomarker Analysis

Single molecular array technology (Simoa; Quanterix Corp) was used to measure serum biomarker levels. Multiplex technology simultaneously quantified UCH-L1, tau, NF-L, and GFAP. Assays were batched to minimize variability, with each batch run with appropriate standards and controls to ensure reliability. To reduce potential batch effects, longitudinal samples from the same individual were run on the same plate, and the 3 groups were distributed randomly across plates. All samples were analyzed in duplicate. In the rare instances in which coefficients of variance exceeded 20%, samples were rerun. Data were not used if intra-assay or interassay performance was above 20%. Samples with concentrations below the level of detection were excluded from analysis. The mean coefficients of variance for each protein were the following for data included in the analyses: 9.02% for UCH-L1, 7.92% for tau, 4.59% for NF-L, and 3.07% for GFAP.

### Statistical Analysis

Statistical analyses were performed using SPSS Statistics, version 24.0 (IBM Corp). Statistically significant results were declared at the nominal significance level of α = .05. Group comparisons of demographic characteristics and clinical outcomes were evaluated using 1-way analyses of variance and χ^2^ tests. Biomarker levels were natural log transformed to decrease the skewness of their distributions. Linear mixed-effects (LME) models were used to evaluate changes in clinical measures and biomarker levels within athletes over time as a function of group, with visit modeled as a repeated factor (ie, baseline, acute postinjury time point, 24-48 hours after injury, point of reporting being asymptomatic, or 7 days after RTP), group (ie, athletes with concussion, contact sport controls, or non–contact sport controls), and the group × visit interaction. Sensitivity analyses were conducted to test whether acute injury severity differed systematically between athletes with and without biomarker data (ie, more severe acute injury in those with collected blood samples), thereby possibly biasing the biomarker results. The SCAT-3 symptom severity was used as an index of acute injury severity. Sensitivity analyses with the covariates of sex, body mass index, and number of prior concussions indicated that these covariates were not significantly associated with the LME results for biomarkers. The LME models assessed biomarker changes over time and between groups in athletes with concussion with and without loss of consciousness (LOC) and/or posttraumatic amnesia (PTA) compared with both control groups. Bonferroni correction to protect the familywise error at α = .05 was conducted for post hoc tests when indicated (eg, after significant interactions).

Receiver operating characteristic curves and area under the curve (AUC) with 95% CIs were used to quantify the ability of markers to discriminate athletes with concussion from contact sport controls or non–contact sport controls at the acute postinjury time point and 24 to 48 hours after injury. Logistic regression analyses were conducted to assess whether the inclusion of blood biomarker levels provided additional information to aid in the classification of athletes with concussion and controls than that provided by self-reported symptoms alone (ie, SCAT-3). A 2-sided *P* < .05 was considered to be statistically significant.

## Results

A total of 264 athletes (mean [SD] age, 19.08 [1.24] years; 211 [79.9%] male) sustained a concussion during the study period and met the study criteria. Similar visits were completed by 138 matched contact sport controls (mean [SD] age, 19.03 [1.27] years; 107 [77.5%] male) and 102 matched non–contact sport controls (mean [SD] age, 19.39 [1.25] years; 82 [80.4%] male). The Table summarizes the sample characteristics for all groups.

**Table.** Sample Characteristics for Athletes With Concussion, Contact Sport Controls, and Non–Contact Sport Controls^a^

Characteristic	Concussion Group (n = 264)	Contact Sport Control Group (n = 138)	Non–Contact Sport Control Group (n = 102)	Test Statistic, *F* or χ^2^^b^	*P* Value
Age, mean (SD), y	19.08 (1.24)	19.03 (1.27)	19.39 (1.25)	2.87	.06
Male	211 (79.9)	107 (77.5)	82 (80.4)	0.40	.82
Height, mean (SD), cm	180.46 (10.49)	180.74 (10.26)	184.08 (9.55)	4.83	.008
Weight, mean (SD), kg	88.07 (21.01)	86.86 (20.08)	79.97 (15.40)	6.34	.002
Length of sport participation, mean (SD), y	10.43 (4.23)	10.85 (3.90)	10.67 (3.66)	0.64	.53
Race					
White	171 (64.8)	93 (67.4)	78 (76.5)	6.38	.17
Black	59 (22.3)	32 (23.2)	13 (12.7)		
Other, unknown, or not reported	34 (12.9)	13 (9.4)	11 (10.8)		
Ethnicity					
Non-Hispanic	218 (82.6)	116 (84.1)	93 (91.2)	NA^c^	.01
Hispanic	16 (6.1)	9 (6.5)	8 (7.8)		
Unknown or not reported	30 (11.4)	13 (9.4)	1 (1.0)		
ADHD	26 (9.7)	10 (7.2)	2 (1.9)	NA^c^	.03
Learning disorder	4 (1.5)	1 (0.7)	2 (1.9)	NA^c^	.69
WTAR standard score, mean (SD)	108.85 (11.82)	109.34 (12.31)	109.54 (12.16)	0.12	.89
No. of prior concussions					
0	146 (56.4)	95 (69.9)	82 (81.2)	NA^c^	<.001
1	81 (31.3)	33 (24.3)	17 (16.8)		
2	21 (8.1)	5 (3.7)	2 (2.0)		
≥3	11 (4.2)	3 (2.2)	0		
Sport					
Football	119 (45.1)	70 (50.7)	0	NA^c^	<.001
Ice hockey	24 (9.1)	10 (7.2)	0		
Lacrosse	18 (6.7)	8 (5.8)	0		
Rugby	49 (18.6)	8 (5.8)	0		
Soccer	47 (17.5)	30 (21.7)	0		
Wrestling	7 (2.6)	2 (1.4)	0		
Baseball	0	0	37 (36.3)		
Basketball	0	0	13 (12.7)		
Cross country or track	0	0	37 (36.3)		
Field event	0	0	8 (7.8)		
Softball	0	0	7 (6.9)		
Other	0	10 (7.2)	0		

Abbreviations: ADHD, attention-deficit/hyperactivity disorder; NA, not applicable; WTAR, Wechsler Adult Reading Test.

^a^ Data are presented as number (percentage) of study participants unless otherwise indicated.

^b^ Data for continuous variables are *F* values and for categorical values are χ^2^.

^c^ The Fisher exact test was used to evaluate the frequency of sample characteristics with fewer than 5 observations in each category (ie, ethnicity, ADHD status, learning disorder status, and number of prior concussions).

### Acute-Phase Clinical Outcomes

Athletes with concussion had significantly elevated SCAT-3 symptom severity scores and performed poorer than both control groups on BESS and SAC at the acute postinjury time point (SCAT-3: concussed vs contact sport control mean difference, 25.97; 95% CI, 23.38 to 28.56; *P* < .001; concussed vs non–contact sport control mean difference, 25.60; 95% CI, 22.76 to 28.43; *P* < .001; BESS: concussed vs contact sport control mean difference, 5.63; 95% CI, 3.86 to 7.41; *P* < .001; concussed vs non–contact sport control mean difference, 4.66; 95% CI, 2.72 to 6.59; *P* < .001; SAC: concussed vs contact sport control mean difference, −1.64; 95% CI, −2.17 to −1.12; *P* < .001; concussed vs non–contact sport control mean difference, −1.67; 95% CI, −2.25 to −1.09; *P* < .001) and 24 to 48 hours after injury (24 hours: SCAT-3: concussed vs contact sport control mean difference, 19.57; 95% CI, 17.08 to 22.06; *P* < .001; concussed vs non–contact sport control mean difference, 19.36; 95% CI, 16.61 to 22.10; *P* < .001; BESS: concussed vs contact sport control mean difference, 4.33; 95% CI, 2.64 to 6.01; *P* < .001; concussed vs non–contact sport control mean difference, 3.15; 95% CI, 1.29 to 5.00; *P* < .001; SAC: concussed vs contact sport control mean difference, −0.84; 95% CI, −1.35 to −0.33; *P* < .001; concussed vs non–contact sport control mean difference, −0.72; 95% CI, −1.27 to −0.16; *P* < .001) (eFigure and eTable 2 in the Supplement).

At 24 to 48 hours after injury, the BSI-18 score was also elevated in athletes with concussion compared with baseline and both control groups (BSI-18 at 24 hours: concussed vs contact sport control mean difference, 3.58; 95% CI, 2.70-4.46; *P* < .001; concussed vs non–contact sport control mean difference, 3.75; 95% CI, 2.78-4.71; *P* < .001). There were no significant differences in the concussion group compared with baseline or controls on the SCAT-3, BESS, SAC, or BSI-18 at the asymptomatic and 7-day post-RTP time points. All significant pairwise comparisons of clinical outcome assessments are presented in eTables 1-3 in the Supplement.

### Acute-Phase Biomarker Levels

Of the 264 athletes with concussion, 243 (92.0%) had blood specimens collected at baseline and acute postinjury time points, and 206 athletes with concussion (78.0%) had blood samples collected at 1 or both of the acute postinjury and 24 to 48 hours after injury time points. Sensitivity analysis demonstrated that athletes with and without blood samples at the acute postinjury time point did not differ in acute injury severity based on SCAT-3 symptom severity score at the acute postinjury or 24- to 48-hour time point. Median and interquartile ranges (IQRs) for time from injury to clinical testing and blood sample collection at each of the postinjury time points were as follows: acute postinjury time point, 3.42 hours (IQR, 1.52-14.08 hours); 24 to 48 hours after injury, 41.25 hours (IQR, 26.13-46.42 hours); point of reporting being asymptomatic, 6.96 days (IQR, 4.06-10.80 days); and 7 days after RTP, 21.41 days (IQR, 16.69-28.16 days).

At preseason baseline, no differences were found between the concussion and contact sport control group in GFAP, UCH-L1, NF-L, or tau. Furthermore, contact sport and non–contact sport controls had stable levels of all biomarkers across all time points, with no significant main effect for visit in either control group and no differences between the 2 control groups on any biomarker at any time point.

**Figure 1.**
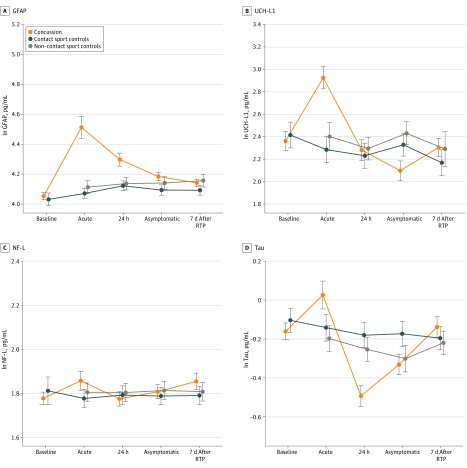
Baseline and Postinjury Biomarker Levels in the Concussion, Contact Sport Control, and Non–Contact Sport Control Groups Biomarker levels represent natural log (ln) transformed scale. Error bars indicate SEs. GFAP indicates glial fibrillary acidic protein; NF-L, neurofilament light chain; RTP, return to play; and UCH-L1, ubiquitin C-terminal hydrolase-L1.

Figure 1 presents the profile of biomarker levels for athletes with concussion and control athletes at the baseline and postinjury time points (eTable 4 in the Supplement also presents additional nontransformed quantitative data on biomarker characteristics by group across all time points). Longitudinally, a significant interaction (group × visit) was found for GFAP (*F*_7,1507.36_ = 16.18, *P* < .001), UCH-L1 (*F*_7,1153.09_ = 5.71, *P* < .001), and tau (*F*_7,1480.55_ = 6.81, *P* < .001); the interaction for NF-L was not significant (*F*_7,1506.90_ = 1.33, *P* = .23).

Post hoc analysis revealed that athletes with concussion had a significant elevation in GFAP levels at all postinjury time points compared with baseline (acute postinjury time point: mean difference, 0.430 pg/mL; 95% CI, 0.339-0.521 pg/mL; *P* < .001; 24-48 hours after injury: mean difference, 0.255 pg/mL; 95% CI, 0.183-0.328 pg/mL; *P* < .001; point of reporting being asymptomatic: mean difference, 0.124 pg/mL; 95% CI, 0.056-0.191 pg/mL; *P* < .001; and 7 days after RTP: mean difference, 0.092 pg/mL; 95% CI, 0.022-0.162 pg/mL; *P* = .002) and significantly higher levels of GFAP compared with contact sport and non–contact sport controls at the acute postinjury time point (contact sport controls: mean difference, 0.419 pg/mL; 95% CI, 0.295-0.543 pg/mL; *P* < .001; non–contact sport controls: mean difference, 0.378 pg/mL; 95% CI, 0.242-0.514 pg/mL; *P* < .001) and 24 to 48 hours after injury (contact sport controls: mean difference, 0.191 pg/mL; 95% CI, 0.076-0.306 pg/mL; *P* < .001; non–contact sport controls: mean difference, 0.177 pg/mL; 95% CI, 0.050-0.304 pg/mL; *P* = .003). There were no significant group differences in GFAP levels at the asymptomatic time point or 7 days after RTP.

Athletes with concussion had a significant elevation in UCH-L1 levels compared with baseline (mean difference, 0.449 pg/mL; 95% CI, 0.167-0.732 pg/mL; *P* < .001) and significantly higher UCH-L1 levels than both control groups at the acute postinjury time point (contact sport controls: mean difference, 0.577 pg/mL; 95% CI, 0.236-0.919 pg/mL; *P* < .001; non–contact sport controls: mean difference, 0.463 pg/mL; 95% CI, 0.088-0.839 pg/mL; *P* = .01). There were no elevations from baseline or group differences in UCH-L1 levels at 24 to 48 hours after injury or 7 days after RTP. Athletes with concussion had significantly lower UCH-L1 levels at the asymptomatic time point compared with baseline (mean difference, −0.321 pg/mL; 95% CI, −0.546 to −0.095 pg/mL; *P* < .001) and compared with non–contact sport controls (concussed vs non–contact sport controls: mean difference, −0.373 pg/mL; 95% CI, −0.714 to −0.032 pg/mL; *P* = .03).

For tau level, athletes with concussion had significant elevation from baseline (mean difference, 0.221 pg/mL; 95% CI, 0.046-0.396 pg/mL; *P* = .004) and significantly higher tau levels than both control groups at the acute postinjury time point (contact sport controls: mean difference, 0.230 pg/mL; 95% CI, 0.020-0.439 pg/mL; *P* = .03; non–contact sport controls, mean difference, 0.266 pg/mL; 95% CI, 0.038-0.493 pg/mL; *P* = .02). At 24 to 48 hours after injury, tau levels in athletes with concussion were significantly lower than baseline levels (mean difference, −0.320 pg/mL; 95% CI, −0.461 to −0.178 pg/mL; *P* < .001) and levels in both control groups (contact sport controls: mean difference, −0.285 pg/mL; 95% CI, −0.475 to −0.094 pg/mL; *P* = .001; non–contact sport controls: mean difference, −0.217 pg/mL; 95% CI, −0.425 to −0.009 pg/mL; *P* = .04). No group differences were evident at other postinjury time points. All significant pairwise comparisons of biomarkers are presented in eTable 5 and eTable 6 in the Supplement. In our overall analysis, there was no significant interaction or main effects for group or visit on NF-L.

**Figure 2.**
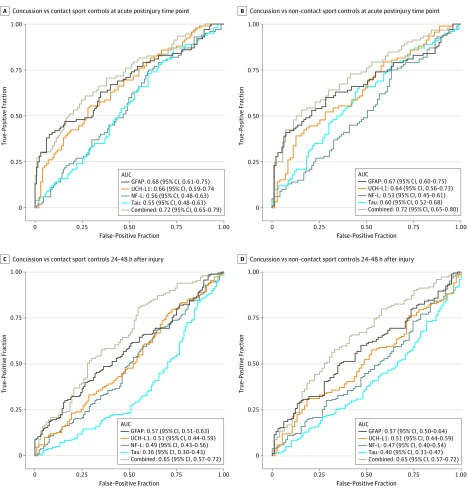
Receiver Operating Characteristic Curve Results for Biomarkers at the Acute Postinjury Time Point and 24 to 48 Hours After Injury AUC indicates area under the curve; GFAP, glial fibrillary acidic protein; NF-L, neurofilament light chain; and UCH-L1, ubiquitin C-terminal hydrolase-L1.

Figure 2 and eTable 7 in the Supplement provide results from receiver operating characteristic curves and AUC statistics for assessment of the use of GFAP, UCH-L1, NF-L, and tau levels to discriminate athletes with concussion from contact sport and non–contact sport controls at the acute postinjury and 24 to 48 hours after injury time points. Across the individual biomarkers, AUC statistics ranged from 0.53 to 0.68 at the acute postinjury time point and were considerably lower at 24 to 48 hours after injury. The highest acute postinjury AUC values were for GFAP (AUC, 0.68; 95% CI, 0.61-0.75; *P* < .001 vs contact sport controls; AUC, 0.67; 95% CI, 0.60-0.75; *P* < .001 vs non–contact sport controls) and UCH-L1 (AUC, 0.66; 95% CI, 0.59-0.74; *P* < .001 vs contact sport controls; AUC, 0.64; 95% CI, 0.56-0.73; *P* = .002 vs non–contact sport controls). The AUC for the combination of GFAP and UCH-L1 at the acute postinjury time point ranged from 0.70 (95% CI, 0.62-0.78; *P* < .001 vs non–contact sport controls) to 0.71 (95% CI, 0.64-0.78; *P* < .001 vs contact sport controls), whereas the AUC for all 4 biomarkers combined was 0.72 (95% CI, 0.65-0.79; *P* < .001 vs contact sport controls; 95% CI, 0.65-0.80; *P* < .001 vs non–contact sport controls). The AUC for SCAT-3 at the acute postinjury time point ranged from 0.94 (95% CI, 0.91-0.97; *P* < .001 vs non–contact sport controls) to 0.95 (95% CI, 0.92-0.97; *P* < .001 vs contact sport controls). In comparison, the AUC was 0.65 (95% CI, 0.59-0.71; *P* < .001 vs contact sport controls; 95% CI, 0.59-0.71; *P* < .001 vs non–contact sport controls) for SAC and ranged from 0.64 (95% CI, 0.57-0.71; *P* < .001 vs non–contact sport controls) to 0.68 (95% CI, 0.62-0.74; *P* < .001 vs contact sport controls) for BESS at the acute postinjury time point.

Logistic regression analysis revealed that inclusion of GFAP was associated with greater classification of athletes with concussion from both contact sport controls (β = 12.298; 95% CI, 2.776-54.481; *P* = .001) and non–contact sport controls (β = 5.438; 95% CI, 1.676-17.645; *P* = .005) than SCAT-3 symptom severity score alone at the acute postinjury time point.

### Association of LOC and PTA With Biomarker Levels

**Figure 3.**
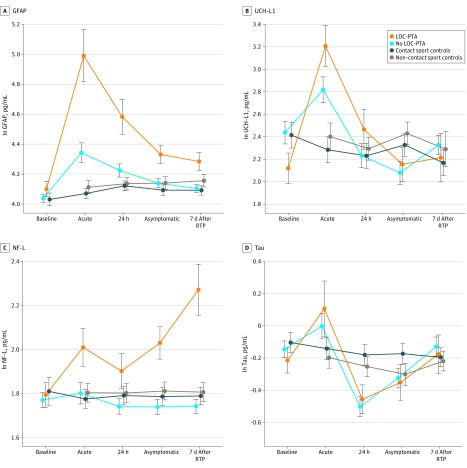
Baseline and Postinjury Biomarker Levels in Athletes With Concussion With Either Loss of Consciousness (LOC) or Posttraumatic Amnesia (PTA) (LOC-PTA), Athletes With Concussion With No LOC or PTA (No LOC-PTA), Contact Sport Controls, and Non–Contact Sport Controls Biomarker levels represent natural log (ln) transformed scale. Error bars indicate SEs. GFAP indicates glial fibrillary acidic protein; NF-L, neurofilament light chain; RTP, return to play; and UCH-L1, ubiquitin C-terminal hydrolase-L1.

Of the 264 athletes with concussion, 207 (78.4%) had neither LOC nor PTA associated with their injury (no LOC-PTA); 57 athletes (21.6%) had PTA or LOC, including 40 athletes (15.2%) who experienced PTA without LOC and 17 (6.4%) who had both LOC and PTA. Athletes with either LOC or PTA (LOC-PTA) were combined in our analysis because of the low number of athletes with LOC. Figure 3 presents results from our analysis comparing LOC-PTA, no LOC-PTA, contact sport control, and non–contact sport control groups. Nontransformed biomarker characteristics of the LOC and PTA subgroups are reported in eTable 8 in the Supplement.

The LME analysis revealed a significant interaction of group and visit across all biomarkers (GFAP: *F*_11,1509.57_ = 16.14, *P* < .001; UCH-L1: *F*_11,1154.18_ = 4.77, *P* < .001; NF-L: *F*_11,1511.84_ = 4.71, *P* < .001; tau: *F*_11,1486.61_ = 4.46, *P* < .001). There were no significant differences between the LOC and PTA subgroups and contact sport controls at baseline on any biomarkers. The no LOC-PTA group had higher GFAP levels compared with both control groups (contact sport controls: mean difference, 0.274 pg/mL; 95% CI, 0.130-0.418 pg/mL; *P* < .001; non–contact sport controls: mean difference, 0.233 pg/mL; 95% CI, 0.077-0.389 pg/mL; *P* < .001) and UCH-L1 (mean difference, 0.518 pg/mL; 95% CI, 0.112-0.924 pg/mL; *P* = .005) compared with contact sport controls at the acute postinjury time point.

The LOC-PTA group had significantly elevated GFAP levels compared with baseline at the acute postinjury time point (mean difference, 0.803 pg/mL; 95% CI, 0.625-0.982 pg/mL; *P* < .001), 24 to 48 hours after injury (mean difference, 0.481 pg/mL; 95% CI, 0.326-0.637 pg/mL; *P* < .001), and at the asymptomatic time point (mean difference, 0.212 pg/mL; 95% CI, 0.074-0.350 pg/mL; *P* < .001). The GFAP levels in the LOC-PTA group were also significantly higher than those in the no LOC-PTA, contact sport control, and non–contact sport control groups at the acute postinjury time point (no LOC-PTA: mean difference, 0.583 pg/mL; 95% CI, 0.369-0.797 pg/mL; *P* < .001; contact sport controls: mean difference, 0.857 pg/mL; 95% CI, 0.647-1.068 pg/mL; *P* < .001; non–contact sport controls: mean difference, 0.816 pg/mL; 95% CI, 0.597-1.035 pg/mL; *P* < .001), 24 to 48 hours after injury (no LOC-PTA: mean difference, 0.368 pg/mL; 95% CI, 0.179-0.558 pg/mL; *P* < .001; contact sport controls: mean difference, 0.482 pg/mL; 95% CI, 0.287-0.676 pg/mL; *P* < .001; non–contact sport controls: mean difference, 0.468 pg/mL; 95% CI, 0.264-0.671 pg/mL; *P* < .001), and asymptomatic time point (no LOC-PTA: mean difference, 0.196 pg/mL; 95% CI, 0.022-0.371 pg/mL; *P* = .02; contact sport controls: mean difference, 0.253 pg/mL; 95% CI, 0.070-0.435 pg/mL; *P* = .002; non–contact sport controls: mean difference, 0.193 pg/mL; 95% CI, 0.001-0.385 pg/mL; *P* = .048).

The secondary analysis rendered markedly different results for NF-L. In the LOC-PTA group, there was an increase in NF-L levels over time after injury. The LOC-PTA group had significantly higher NF-L levels than the no LOC-PTA group (mean difference, 0.290 pg/mL; 95% CI, 0.097-0.482 pg/mL; *P* < .001) and the contact sport control group (mean difference, 0.248 pg/mL; 95% CI, 0.047-0.449 pg/mL; *P* = .007) at the asymptomatic time point. This elevation in NF-L levels in the LOC-PTA group persisted through 7 days after RTP compared with the no LOC-PTA group (mean difference, 0.498 pg/mL; 95% CI, 0.295-0.701 pg/mL; *P* < .001) and both control groups (contact sport controls: mean difference, 0.481 pg/mL; 95% CI, 0.271-0.692 pg/mL; *P* < .001; non–contact sport controls: mean difference, 0.448 pg/mL; 95% CI, 0.228-0.668 pg/mL; *P* < .001). All significant pairwise comparisons of biomarkers in athletes with and without LOC and PTA are presented in eTable 9 and eTable 10 in the Supplement.

**Figure 4.**
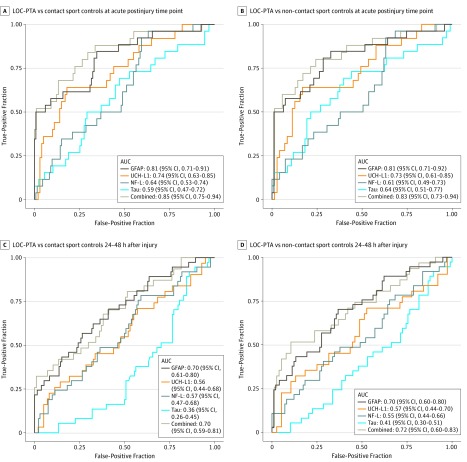
Receiver Operating Characteristic Curve Results for Biomarkers Differentiating Athletes With Concussion With Either Loss of Consciousness (LOC) or Posttraumatic Amnesia (PTA) (LOC-PTA) From Contact Sport Controls and Non–Contact Sport Controls at the Acute Postinjury Time Point and 24 to 48 Hours After Injury AUC indicates area under the curve; GFAP, glial fibrillary acidic protein; NF-L, neurofilament light chain; and UCH-L1, ubiquitin C-terminal hydrolase-L1.

Figure 4 and eTable 11 in the Supplement provide AUC statistics for the biomarkers discriminating among LOC-PTA, no LOC-PTA, and contact sport and non–contact sport controls at the acute postinjury time point and 24 to 48 hours after injury. In parallel to the higher biomarker levels in the LOC-PTA group, the highest AUC was for differentiating the LOC-PTA group from the no LOC-PTA group and both controls groups. The AUC for the full biomarker set differentiating the LOC-PTA group from the no LOC-PTA group (AUC, 0.73; 95% CI, 0.59-0.83; *P* < .001) at the acute postinjury time point was equivalent to the AUC that differentiated all athletes with concussion from both control groups (AUC, 0.72; 95% CI, 0.65-0.79; *P* < .001 vs contact sport controls; AUC, 0.72; 95% CI, 0.65-0.80; *P* < .001 vs non–contact sport controls). In differentiating the LOC-PTA group from the control athletes without concussion at the acute postinjury time point, the AUC was 0.81 for GFAP (95% CI, 0.71-0.91; *P* < .001 vs contact sport controls; 95% CI, 0.71-0.92; *P* < .001 vs non–contact sport controls) and ranged from 0.73 (95% CI, 0.61-0.85; *P* < .001 vs non–contact sport controls) to 0.74 (95% CI, 0.63-0.85; *P* < .001 vs contact sport controls) for UCH-L1. The AUC for the combination of GFAP and UCH-L1 at the acute postinjury time point was 0.84 (95% CI, 0.74-0.94; *P* < .001 vs contact sport controls; 95% CI, 0.73-0.94; *P* < .001 vs non–contact sport controls), and the AUC for all 4 biomarkers combined ranged from 0.83 (95% CI, 0.73-0.94; *P* < .001 vs non–contact sport controls) to 0.85 (95% CI, 0.75-0.94; *P* < .001 vs contact sport controls). At the acute postinjury time point, the SCAT-3 symptom score was not different between the athletes with LOC-PTA and no LOC-PTA (AUC, 0.54; 95% CI, 0.45-0.64; *P* = .40).

## Discussion

In this prospective study of blood biomarker levels after SRC, athletes with concussion had significant elevations in GFAP, UCH-L1, and tau levels compared with preseason baseline levels and compared with 2 groups of control athletes without concussion during the acute postinjury period. Elevations acute postinjury time point were most evident in GFAP and UCH-L1, 2 biomarkers recently granted US Food and Drug Administration clearance for clinical use in identifying the presence of underlying intracranial injury after more severe TBI. We also saw incrementally higher elevations in GFAP, UCH-L1, and NF-L levels after more severe SRC that involved LOC and PTA. The GFAP level remained elevated in athletes with concussion up to 24 to 48 hours after injury, with even more extended elevations in GFAP and NF-L levels for several days after concussion with LOC and PTA. Overall, these findings provide further support for the role of biomarkers in the study of concussion as well as the eventual prospect of potential clinical utility.

Data from the current study potentially extend the lower threshold of the TBI biomarker sensitivity by detecting a signal on GFAP and UCH-L1 after acute SRC, arguably the mildest of injuries along the TBI spectrum. These findings are particularly relevant to a subset of patients with mild TBI who are not typically treated at level I trauma centers, such as athletes affected by SRC and military service members with mild TBI. The potential role of blood biomarkers in SRC is accentuated by the fact that neuroimaging study results are normal in most athletes with acute concussion.^17^ Although significantly higher than baseline and in the 2 control groups, biomarker levels in this study were considerably below levels reported in studies of moderate and severe TBI. The findings of higher levels of GFAP and UCH-L1 as well as increasing levels of NF-L after SRC with LOC and PTA are closer to the pattern observed in a previous study^9^ of complicated mild or moderate TBI. Collectively, our findings in athletes with LOC and PTA, often considered an indicator of more severe injury, suggest a dose response of TBI severity to candidate TBI biomarker levels, which could be particularly valuable for triaging patients with TBI in critical care settings and stratifying patients for TBI clinical trials.

From a clinical standpoint, candidate biomarkers in the current study had a modest ability to differentiate athletes with concussion from control athletes in the acute phase. The AUC at the initial acute postinjury time point was 0.70 to 0.71 for the combination of GFAP and UCH-L1 and 0.72 for the 4-plex of biomarkers, which was slightly higher than SAC (AUC, 0.65) and BESS (AUC, 0.64-0.68). Perhaps more important in terms of both research and clinical utility is the finding that, beyond SCAT-3 symptom severity score, GFAP at the acute postinjury time point was associated with greater classification of athletes with concussion, contact sport controls, and non–contact sport controls. This finding suggests that biomarkers may provide an objective and independent means to increase diagnostic certainty without the dilemma of clinical diagnosis based on subjective, self-reported symptoms that are not specific to concussion. The potential prognostic utility of biomarkers in assessing recovery after SRC also warrants further investigation.

### Strengths and Limitations

Several methodologic attributes of the current study strengthen our results. The availability of preinjury baseline blood and serial postinjury blood sample collection is a unique advantage of the SRC research model that strengthens the interpretation of findings from the current study. The collection of blood biomarker levels soon after injury (median, 3.42 hours after injury) was also critical to biomarker signal detection in our study, whereas longitudinal follow-up of athletes with concussion and control athletes was critical to understanding the trajectory and time course of dynamic biomarker abnormalities during clinical and neurobiological recovery after SRC. An important finding that has relevance to the broader landscape of TBI biomarker research is that contact sport and non–contact sport controls in our study had stable levels of all biomarkers, with no association of time or contact sport exposure with any biomarker at any time point.

This study has limitations. Our sample of athletes with concussion was confined to collegiate athletes participating in high-contact sports, such as football, soccer, lacrosse, ice hockey, rugby, and wrestling. Further study of concussion biomarkers in other sports would be beneficial. Our study did not include athletes at different developmental stages or competitive levels (eg, high school or professional), although a study^11^ of biomarkers in high school athletes reported similar results. As is common in longitudinal studies with several data collection points, we encountered some degree of missingness across visits in all 3 groups. Sensitivity analysis demonstrated, however, that athletes with and without blood samples did not differ in acute injury severity, suggesting that our primary biomarker results were not systematically biased by the effects of missing data (ie, more severe acute injury in those with collected blood samples). Also, our group comparisons of biomarker results do not equate to diagnostic certainty at the individual case level, as would be required to support any recommendations for clinical use of biomarkers in concussion assessment and management.

## Conclusions

This study’s findings provide support for the utility of blood biomarkers as research tools to inform the underlying pathophysiological mechanisms of concussion, but there is still not sufficient evidence to support broad implementation of biomarkers for clinical diagnosis, evaluation, and management of SRC. The goal of biomarkers should not be to replace signs and symptoms in clinical diagnosis of concussion but rather to augment clinical specificity and confidence with more objective indicators of brain injury. Further research is required to optimize and validate a multiplex of biomarkers that would meet accepted standards for clinical adoption. In addition, if supported by the evidence of clinical use, there is a critical need for a translational pathway for biomarker implementation in acute TBI and concussion care, including point-of-care testing of patients with concussion or TBI.

## Notes

Supplement. **eFigure.** Baseline and Postinjury SCAT, BESS, SAC and BSI-18 Data for Concussed, Contact Control, and Non-Contact Control Athletes **eTable 1.** Clinical Outcome Assessment Characteristics for Concussed, Contact Control and Non-Contact Control Athletes at Baseline, Acute Post-Injury, and 24-48 Hours Post-Injury Time Points **eTable 2.** Within-Subjects Comparisons of Clinical Outcome Assessments **eTable 3.** Between-Subjects Comparisons of Clinical Outcome Assessments **eTable 4.** Biomarker Characteristics for Concussed, Contact Control, and Non-Contact Control Athletes at All Time Points **eTable 5.** Within-Subjects Comparisons of Biomarkers in Concussed Athletes **eTable 6.** Between-Subjects Comparisons of Biomarkers in Concussed, Contact Control, and Non-Contact Control Athletes **eTable 7.** Area Under the Curve (AUC) Summaries for Biomarkers SAC and BESS at Acute Post-Injury and 24-48 Hours Post-Injury Time Points **eTable 8.** Biomarker Characteristics for Concussed Athletes With or Without Loss of Consciousness or Post-Traumatic Amnesia at All Time Points **eTable 9.** Within-Subjects Comparisons of Biomarkers in Athletes With or Without Loss of Consciousness or Post-Traumatic Amnesia **eTable 10.** Between-Subjects Comparisons of Biomarkers in Athletes With or Without Loss of Consciousness or Post-Traumatic Amnesia, Contact Control, and Non-Contact Control Athletes **eTable 11.** Subgroup Area Under the Curve (AUC) for Biomarkers SAC and BESS at Acute Post-Injury and 24-48 Hours Post-Injury Time Points
