# Supplementary material for: Association of Blood Biomarkers With Acute Sport-Related Concussion in Collegiate Athletes: Findings From the NCAA and Department of Defense CARE Consortium
Source: JAMA Netw Open. 2020 Jan 24;3(1):e1919771. doi: 10.1001/jamanetworkopen.2019.19771 (PMC6991302; doi:10.1001/jamanetworkopen.2019.19771)
Supplement: Supplement. — eFigure. Baseline and Postinjury SCAT, BESS, SAC and BSI-18 Data for Concussed, Contact Control, and Non-Contact Control Athletes eTable 1. Clinical Outcome Assessment Characteristics for Concussed, Contact Control and Non-Contact Control Athletes at Baseline, Acute Post-Injury, and 24-48 Hours Post-Injury Time Points eTable 2. Within-Subjects Comparisons of Clinical Outcome Assessments eTable 3. Between-Subjects Comparisons of Clinical Outcome Assessments eTable 4. Biomarker Characteristics for Concussed, Contact Control, and Non-Contact Control Athletes at All Time Points eTable 5. Within-Subjects Comparisons of Biomarkers in Concussed Athletes eTable 6. Between-Subjects Comparisons of Biomarkers in Concussed, Contact Control, and Non-Contact Control Athletes eTable 7. Area Under the Curve (AUC) Summaries for Biomarkers SAC and BESS at Acute Post-Injury and 24-48 Hours Post-Injury Time Points eTable 8. Biomarker Characteristics for Concussed Athletes With or Without Loss of Consciousness or Post-Traumatic Amnesia at All Time Points eTable 9. Within-Subjects Comparisons of Biomarkers in Athletes With or Without Loss of Consciousness or Post-Traumatic Amnesia eTable 10. Between-Subjects Comparisons of Biomarkers in Athletes With or Without Loss of Consciousness or Post-Traumatic Amnesia, Contact Control, and Non-Contact Control Athletes eTable 11. Subgroup Area Under the Curve (AUC) for Biomarkers SAC and BESS at Acute Post-Injury and 24-48 Hours Post-Injury Time Points [file jamanetwopen-3-e1919771-s001.pdf]

Supplementary Online Content

McCrea M, Broglio SP, McAllister TW, et al; CARE Consortium Investigators. Association of blood biomarkers with acute sport-related concussion in collegiate athletes: findings from the NCAA and Department of Defense CARE Consortium. *JAMA Netw Open*. 2020;3(1):e1919771. doi:10.1001/jamanetworkopen.2019.19771

eFigure. Baseline and Postinjury SCAT, BESS, SAC and BSI-18 Data for Concussed, Contact Control, and Non-Contact Control Athletes

eTable 1. Clinical Outcome Assessment Characteristics for Concussed, Contact Control and Non-Contact Control Athletes at Baseline, Acute Post-Injury, and 24-48 Hours Post-Injury Time Points

eTable 2. Within-Subjects Comparisons of Clinical Outcome Assessments

eTable 3. Between-Subjects Comparisons of Clinical Outcome Assessments

eTable 4. Biomarker Characteristics for Concussed, Contact Control, and Non-Contact Control Athletes at All Time Points

eTable 5. Within-Subjects Comparisons of Biomarkers in Concussed Athletes

eTable 6. Between-Subjects Comparisons of Biomarkers in Concussed, Contact Control, and Non-Contact Control Athletes

eTable 7. Area Under the Curve (AUC) Summaries for Biomarkers SAC and BESS at Acute Post-Injury and 24-48 Hours Post-Injury Time Points

eTable 8. Biomarker Characteristics for Concussed Athletes With or Without Loss of Consciousness or Post-Traumatic Amnesia at All Time Points

eTable 9. Within-Subjects Comparisons of Biomarkers in Athletes With or Without Loss of Consciousness or Post-Traumatic Amnesia

eTable 10. Between-Subjects Comparisons of Biomarkers in Athletes With or Without Loss of Consciousness or Post-Traumatic Amnesia, Contact Control, and Non-Contact Control Athletes

eTable 11. Subgroup Area Under the Curve (AUC) for Biomarkers SAC and BESS at Acute Post-Injury and 24-48 Hours Post-Injury Time Points

This supplementary material has been provided by the authors to give readers additional information about their work.

© 2020 McCrea M et al. *JAMA Network Open*.

eFigure. Baseline and Postinjury SCAT, BESS, SAC and BSI-18 Data for Concussed, Contact Control, and Non-Contact Control Athletes

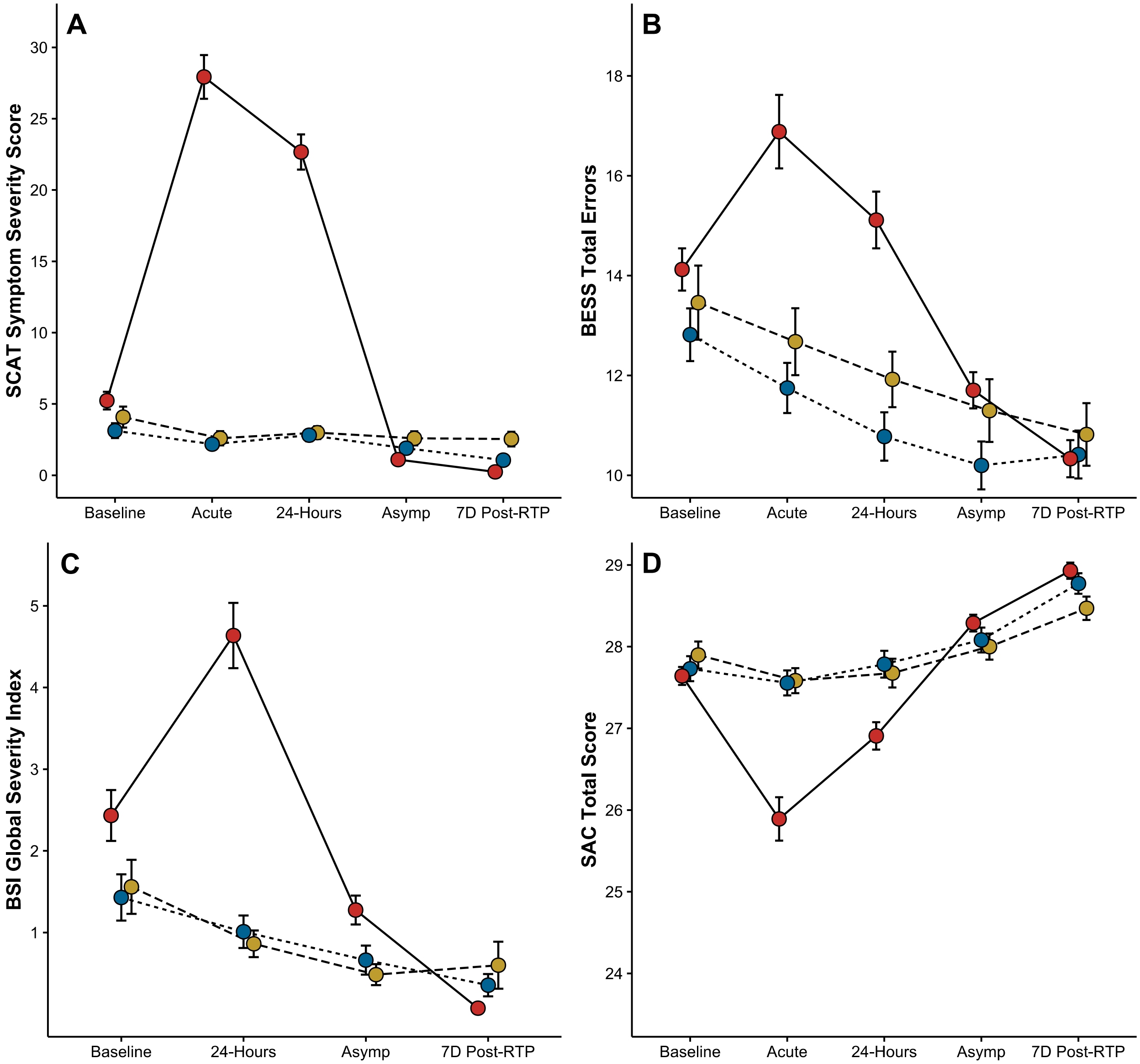

SCAT=Sport Concussion Assessment Tool; BESS=Balance Error Scoring System; BSI=Brief Symptom Inventory-18; SAC=Standardized Assessment of Concussion; Asymp=point at which athlete asymptomatic and return to play protocol initiated; 7D Post-RTP=7 days after unrestricted return to play; for SCAT, BESS and BSI, higher score is worse; for SAC, lower score is worse.

Error bars represent +/- one standard error

© 2020 McCrea M et al. *JAMA Network Open*.

eTable 1. Clinical Outcome Assessment Characteristics for Concussed, Contact Control and Non-Contact Control Athletes at Baseline, Acute Post-Injury, and 24-48 Hours Post-Injury Time Points

		Concussed			Contact Control			Non-Contact Control		
		n	Mean	SD	n	Mean	SD	n	Mean	SD
Baseline	SCAT	258	5.24	9.93	135	3.13	6.03	102	4.08	7.45
	SAC	251	27.64	1.73	137	27.73	1.79	98	27.90	1.63
	BESS	251	14.12	6.69	136	12.82	6.16	96	13.46	7.27
	BSI-18	254	2.43	4.97	135	1.43	3.29	100	1.56	3.31
Acute Post-injury	SCAT	181	27.93	20.60	135	2.18	3.81	101	2.59	5.03
	SAC	175	25.89	3.51	135	27.56	1.78	101	27.58	1.54
	BESS	145	16.88	8.87	135	11.75	5.84	102	12.68	6.77
24-48 Hours Post-injury	SCAT	224	10.39	5.82	135	1.93	3.18	101	2.10	2.73
	SAC	224	22.67	18.42	135	2.81	4.79	101	2.99	4.52
	BESS	216	26.91	2.47	135	27.79	1.91	102	27.68	1.78
	BSI-18	209	4.64	5.78	135	1.01	2.30	102	0.86	1.65
Asymptomatic	SCAT	246	1.10	2.58	133	1.89	3.65	98	2.59	4.87
	SAC	239	28.29	1.58	134	28.08	1.76	99	28.00	1.58
	BESS	239	11.70	5.62	132	10.20	5.51	98	11.30	6.22
	BSI-18	240	1.28	2.74	134	0.66	2.05	99	0.48	1.28
7 Days Post-RTP	SCAT	201	0.24	1.17	131	1.06	2.27	100	2.54	5.14
	SAC	199	28.93	1.39	132	28.77	1.44	100	28.47	1.43
	BESS	196	10.33	5.21	130	10.42	5.47	99	10.82	6.24
	BSI-18	201	0.07	0.40	132	0.36	1.57	100	0.60	2.88

SCAT, Sport Concussion Assessment Tool; SAC, Standardized Assessment of Concussion; BESS, Balance Error Scoring System; BSI-18, Brief Symptom Inventory

Clinical outcome assessments: SCAT symptom severity score, SAC total score, BESS total errors, and BSI-18 Global Severity Index

BSI-18 was not administered at the acute post-injury time point.

© 2020 McCrea M et al. *JAMA Network Open*.

eTable 2. Within-Subjects Comparisons of Clinical Outcome Assessments

			Concussed		Contact Control		Non-Contact Control	
	Time point Comparison		Mean Difference	*P*	Mean Difference	*P*	Mean Difference	*P*
SCAT Symptom Severity Score	Baseline vs.	Acute Post-Injury	-22.854	**<.001**	NS		NS	
		24-48 Hours Post-Injury	-17.073	**<.001**				
		Asymptomatic	4.178	**<.001**				
		7 Days Post-RTP	4.693	**<.001**				
	Acute Post-Injury vs.	24-48 Hours Post-Injury	5.781	**<.001**				
		Asymptomatic	27.031	**<.001**				
		7 Days Post-RTP	27.547	**<.001**				
	24-48 Hours Post-Injury vs.	Asymptomatic	21.251	**<.001**				
		7 Days Post-RTP	21.766	**<.001**				
	Asymptomatic vs.	7 Days Post-RTP	0.516	>.999				
SAC Total Score	Baseline vs.	Acute Post-Injury	1.759	**<.001**	0.190	>.999	0.311	>.999
		24-48 Hours Post-Injury	0.697	**<.001**	-0.071	>.999	0.204	>.999
		Asymptomatic	-0.617	**<.001**	-0.347	.676	-0.108	>.999
		7 Days Post-RTP	-1.133	**<.001**	-1.018	**<.001**	-0.583	.087
	Acute Post-Injury vs.	24-48 Hours Post-Injury	-1.062	**<.001**	-0.261	>.999	-0.107	>.999
		Asymptomatic	-2.376	**<.001**	-0.537	.048	-0.419	.576
		7 Days Post-RTP	-2.892	**<.001**	-1.208	**<.001**	-0.894	**.001**
	24-48 Hours Post-Injury vs.	Asymptomatic	-1.314	**<.001**	-0.276	>.999	-0.312	>.999
		7 Days Post-RTP	-1.830	**<.001**	-0.947	**<.001**	-0.787	**.003**
	Asymptomatic vs.	7 Days Post-RTP	-0.516	**.007**	-0.671	**.005**	-0.475	.319
BESS Total Errors	Baseline vs.	Acute Post-Injury	-3.177	**<.001**	1.147	.366	0.754	>.999
		24-48 Hours Post-Injury	-0.909	.334	2.111	**.001**	1.512	.190
		Asymptomatic	2.423	**<.001**	2.690	**<.001**	2.160	**.009**
		7 Days Post-RTP	3.184	**<.001**	2.452	**<.001**	2.594	**.001**
	Acute Post-Injury vs.	24-48 Hours Post-Injury	2.268	**<.001**	0.964	.800	0.758	>.999
		Asymptomatic	5.600	**<.001**	1.542	.053	1.406	.277
		7 Days Post-RTP	6.361	**<.001**	1.305	.188	1.841	**.039**
	24-48 Hours Post-Injury vs.	Asymptomatic	3.332	**<.001**	0.578	>.999	0.648	>.999
		7 Days Post-RTP	4.093	**<.001**	0.341	>.999	1.082	.900
	Asymptomatic vs.	7 Days Post-RTP	0.761	.865	-0.237	>.999	0.434	>.999
BSI-18 Global Severity Index	Baseline vs.	24-48 Hours Post-Injury	-2.174	**<.001**	0.397	>.999	NS	
		Asymptomatic	1.135	**<.001**	0.769	.260		
		7 Days Post-RTP	2.333	**<.001**	1.077	**.029**		
	24-48 Hours Post-Injury vs.	Asymptomatic	3.309	**<.001**	0.372	>.999		
		7 Days Post-RTP	4.506	**<.001**	0.680	.452		
	Asymptomatic vs.	7 Days Post-RTP	1.197	**<.001**	0.308	>.999		

SCAT, Sport Concussion Assessment Tool; SAC, Standardized Assessment of Concussion; BESS, Balance Error Scoring System; BSI-18, Brief Symptom Inventory; NS, Non-significant simple main effect.
Results are reported only for comparisons with significant simple main effects. BSI-18 was not administered at the acute post-injury time point. All *P*-values are corrected for multiple comparisons (Bonferroni).

© 2020 McCrea M et al. *JAMA Network Open*.

eTable 3. Between-Subjects Comparisons of Clinical Outcome Assessments

	Group Comparison		Baseline		Acute Post-Injury		24-48 Hours Post-Injury	
			Mean Difference	*P*	Mean Difference	*P*	Mean Difference	*P*
SCAT	Concussed vs.	Contact Control	NS		25.969	**<.001**	19.568	**<.001**
		Non-Contact Control			25.597	**<.001**	19.359	**<.001**
	Contact Control vs.	Non-Contact Control			-0.372	>.999	-0.210	>.999
SAC	Concussed vs.	Contact Control	NS		-1.643	**<.001**	-0.843	**<.001**
		Non-Contact Control			-1.669	**<.001**	-0.715	**.007**
	Contact Control vs.	Non-Contact Control			-0.026	>.999	0.128	>.999
BESS	Concussed vs.	Contact Control	NS		5.633	**<.001**	4.329	**<.001**
		Non-Contact Control			4.655	**<.001**	3.145	**<.001**
	Contact Control vs.	Non-Contact Control			-0.978	.752	-1.184	.496
BSI-18	Concussed vs.	Contact Control	1.009	**.014**	.	.	3.581	**<.001**
		Non-Contact Control	0.878	**.078**	.	.	3.746	**<.001**
	Contact Control vs.	Non-Contact Control	-0.132	>.999	.	.	0.166	>.999

SCAT, Sport Concussion Assessment Tool; SAC, Standardized Assessment of Concussion; BESS, Balance Error Scoring System; BSI-18, Brief Symptom Inventory; NS, Non-significant simple main effect.
Results are reported only for comparisons with significant simple main effects. BSI-18 was not administered at the acute post-injury time point. All *P*-values are corrected for multiple comparisons (Bonferroni).

© 2020 McCrea M et al. *JAMA Network Open*.

eTable 4. Biomarker Characteristics for Concussed, Contact Control, and Non-Contact Control Athletes at All Time Points

		Concussed					Contact Control					Non-Contact Control				
		n	Mean	SD	Median	IQR	n	Mean	SD	Median	IQR	n	Mean	SD	Median	IQR
Baseline	GFAP	243	61.81	24.94	57.38	[44.11-75.43]	135	75.05	189.26	56.27	[43.93-68.70]	.	.	.	.	.
	UCH-L1	194	18.88	25.89	11.02	[5.28-26.76]	112	40.07	220.68	11.30	[6.50-22.72]	.	.	.	.	.
	NF-L	243	6.56	3.32	5.88	[4.45-7.75]	135	36.69	349.63	5.90	[4.31-7.97]	.	.	.	.	.
	tau	240	1.04	0.68	0.88	[0.61-1.24]	130	1.20	1.32	0.87	[0.59-1.43]	.	.	.	.	.
Acute Post-injury	GFAP	100	123.97	128.03	76.22	[55.93-128.63]	133	63.40	26.60	58.55	[43.86-79.97]	95	69.45	66.78	61.71	[49.21-73.80]
	UCH-L1	92	27.64	23.73	20.29	[9.36-40.60]	100	18.00	27.33	11.31	[4.95-23.18]	71	17.63	19.48	12.17	[5.44-22.82]
	NF-L	100	7.04	3.31	6.08	[5.10-8.50]	133	6.67	3.71	5.83	[4.31-8.07]	95	6.58	2.60	6.10	[4.81-8.15]
	tau	100	1.34	1.31	1.04	[0.68-1.53]	127	1.12	0.75	0.93	[0.54-1.54]	93	0.99	0.63	0.81	[0.55-1.27]
24-48 Hours Post-injury	GFAP	183	100.75	206.68	67.48	[50.54-94.92]	132	65.94	24.51	60.54	[48.39-80.19]	98	71.33	69.91	59.88	[47.90-83.40]
	UCH-L1	151	15.83	16.49	10.65	[5.60-20.03]	98	16.49	30.10	11.00	[4.50-19.94]	76	14.24	14.91	10.00	[5.22-16.80]
	NF-L	183	6.52	3.22	5.63	[4.43-7.71]	132	6.83	4.54	5.65	[4.55-8.29]	98	6.59	2.71	6.07	[4.58-8.05]
	tau	178	0.78	0.63	0.63	[0.41-0.95]	128	1.05	0.72	0.97	[0.59-1.32]	98	0.93	0.61	0.76	[0.53-1.12]
Asymptomatic	GFAP	231	72.66	41.79	64.85	[48.47-84.64]	130	64.71	26.17	58.75	[44.62-76.58]	98	71.11	67.23	63.03	[49.61-80.07]
	UCH-L1	176	14.43	22.74	9.36	[3.91-17.64]	95	16.70	27.17	11.88	[4.96-19.41]	76	16.16	16.19	11.93	[7.60-20.24]
	NF-L	231	7.05	6.02	5.98	[4.34-8.00]	130	6.67	3.79	6.01	[4.56-7.49]	98	6.62	2.63	6.17	[4.68-8.10]
	tau	227	0.97	0.91	0.76	[0.47-1.12]	130	1.07	0.73	0.89	[0.57-1.44]	94	0.91	0.67	0.72	[0.55-1.19]
7 Days Post-RTP	GFAP	201	66.93	25.32	61.63	[49.20-79.93]	127	63.76	23.07	58.28	[46.11-78.16]	95	72.42	70.43	62.56	[50.92-79.58]
	UCH-L1	160	15.59	14.68	10.75	[5.07-22.99]	95	14.57	19.78	9.17	[5.88-16.20]	73	16.55	16.34	11.40	[6.58-21.94]
	NF-L	201	7.62	6.28	6.11	[4.35-8.12]	127	6.69	3.73	5.85	[4.56-7.81]	95	6.64	2.83	6.21	[4.48-8.15]
	tau	200	1.28	2.43	0.91	[0.54-1.28]	124	1.00	0.65	0.83	[0.57-1.32]	92	0.96	0.70	0.79	[0.53-1.16]

GFAP: glial fibrillary acidic protein; UCH-L1: ubiquitin C-terminal hydrolase-L1; NF-L: neurofilament light; SD: standard deviation; IQR: interquartile range; RTP: return-to-play

Units represent pg/mL for all biomarkers. Blood was not collected from non-contact control athletes at the baseline time point.

GFAP: limit of detection (LOD) = 0.221 pg/mL, lower limit of quantification (LLOQ) = 0.467 pg/mL, upper limit of quantification (ULOQ) = 850 pg/mL; UCH-L1: LOD = 1.74 pg/mL, LLOQ = 5.45 pg/mL, ULOQ = 8855 pg/mL; NF-L: LOD = 0.104 pg/mL, LLOQ = 0.241 pg/mL, ULOQ = 429 pg/mL; tau: LOD = 0.024 pg/mL, LLOQ = 0.053 pg/mL, ULOQ = 84.9 pg/mL

© 2020 McCrea M et al. *JAMA Network Open*.

eTable 5. Within-Subjects Comparisons of Biomarkers in Concussed Athletes

			Concussed	
	Time point Comparison		Mean Difference[a]	*P*
GFAP	Baseline vs.	Acute Post-Injury	-0.430	**<.001**
		24-48 Hours Post-Injury	-0.255	**<.001**
		Asymptomatic	-0.124	**<.001**
		7 Days Post-RTP	-0.092	**.002**
	Acute Post-Injury vs.	24-48 Hours Post-Injury	0.175	**<.001**
		Asymptomatic	0.306	**<.001**
		7 Days Post-RTP	0.338	**<.001**
	24-48 Hours Post-Injury vs.	Asymptomatic	0.132	**<.001**
		7 Days Post-RTP	0.164	**<.001**
	Asymptomatic vs.	7 Days Post-RTP	0.032	>.999
UCH-L1	Baseline vs.	Acute Post-Injury	-0.449	**<.001**
		24-48 Hours Post-Injury	0.127	>.999
		Asymptomatic	0.321	**.001**
		7 Days Post-RTP	0.079	>.999
	Acute Post-Injury vs.	24-48 Hours Post-Injury	-0.576	**<.001**
		Asymptomatic	0.194	.245
		7 Days Post-RTP	-0.048	>.999
	24-48 Hours Post-Injury vs.	Asymptomatic	0.194	.245
		7 Days Post-RTP	-0.048	>.999
	Asymptomatic vs.	7 Days Post-RTP	-0.242	**.044**
NF-L	Baseline vs.	Acute Post-Injury	-0.068	.671
		24-48 Hours Post-Injury	-0.019	>.999
		Asymptomatic	-0.019	>.999
		7 Days Post-RTP	-0.092	**.014**
	Acute Post-Injury vs.	24-48 Hours Post-Injury	0.049	>.999
		Asymptomatic	0.049	>.999
		7 Days Post-RTP	-0.024	>.999
	24-48 Hours Post-Injury vs.	Asymptomatic	0.000	>.999
		7 Days Post-RTP	-0.073	.209
	Asymptomatic vs.	7 Days Post-RTP	-0.073	.125
tau	Baseline vs.	Acute Post-Injury	-0.221	**.004**
		24-48 Hours Post-Injury	0.320	**<.001**
		Asymptomatic	0.184	**.001**
		7 Days Post-RTP	-0.017	>.999
	Acute Post-Injury vs.	24-48 Hours Post-Injury	0.541	**<.001**
		Asymptomatic	0.405	**<.001**
		7 Days Post-RTP	0.204	**.015**
	24-48 Hours Post-Injury vs.	Asymptomatic	-0.136	.079
		7 Days Post-RTP	-0.337	**<.001**
	Asymptomatic vs.	7 Days Post-RTP	-0.200	**<.001**

[a]Mean differences of natural log-transformed biomarker values.
Results are reported only for comparisons with significant simple main effects. All *P*-values are corrected for multiple comparisons (Bonferroni).

© 2020 McCrea M et al. *JAMA Network Open*.

eTable 6. Between-Subjects Comparisons of Biomarkers in Concussed, Contact Control, and Non-Contact Control Athletes

			Acute Post-Injury		24-48 Hours Post-Injury		Asymptomatic	
	Group Comparison		Mean Difference[a]	*P*	Mean Difference	*P*	Mean Difference	*P*
GFAP	Concussed vs.	Contact Control	0.419	**<.001**	0.191	**<.001**	0.100	.105
		Non-Contact Control	0.378	**<.001**	0.177	**.003**	0.040	>.999
	Contact Control vs.	Non-Contact Control	-0.041	>.999	-0.014	>.999	-0.060	.909
UCH-L1	Concussed vs.	Contact Control	0.577	**<.001**	0.023	>.999	-0.200	.375
		Non-Contact Control	0.463	**.010**	-0.034	>.999	-0.373	**.027**
	Contact Control vs.	Non-Contact Control	-0.114	>.999	-0.057	>.999	-0.172	.839
NF-L	Concussed vs.	Contact Control	0.072	.628	0.012	>.999	0.023	>.999
		Non-Contact Control	0.039	>.999	-0.007	>.999	-0.026	>.999
	Contact Control vs.	Non-Contact Control	-0.033	>.999	-0.019	>.999	-0.048	>.999
tau	Concussed vs.	Contact Control	0.230	**.026**	-0.285	**.001**	-0.153	.139
		Non-Contact Control	0.266	**.016**	-0.217	**.038**	-0.037	>.999
	Contact Control vs.	Non-Contact Control	0.036	>.999	0.067	>.999	0.116	.665

[a]Mean differences of natural log-transformed biomarker values.
Results are reported only for comparisons with significant simple main effects. Mean differences All *P*-values are corrected for multiple comparisons (Bonferroni).

© 2020 McCrea M et al. *JAMA Network Open*.

eTable 7. Area Under the Curve (AUC) Summaries for Biomarkers SAC and BESS at Acute Post-Injury and 24-48 Hours Post-Injury Time Points

		Concussed vs. Contact Controls			Concussed vs. Non-Contact Controls		
		AUC	95% CI	P^a	AUC	95% CI	*P*
Acute Post-injury	GFAP	0.68	[0.61, 0.75]	**<.001**	0.67	[0.60, 0.75]	**<.001**
	UCH-L1	0.66	[0.59, 0.74]	**<.001**	0.64	[0.56, 0.73]	**.002**
	NF-L	0.56	[0.48, 0.63]	.152	0.53	[0.45, 0.61]	.521
	tau	0.55	[0.48, 0.63]	.196	0.60	[0.52, 0.68]	**.021**
	GFAP+UCH-L1	0.71	[0.64, 0.78]	**<.001**	0.70	[0.62, 0.78]	**<.001**
	All Biomarkers	0.72	[0.65, 0.79]	**<.001**	0.72	[0.65, 0.80]	**<.001**
	SAC	0.65	[0.59, 0.71]	**<.001**	0.65	[0.59, 0.71]	**<.001**
	BESS	0.68	[0.62, 0.74]	**<.001**	0.64	[0.57, 0.71]	**<.001**
	SCAT3	0.95	[0.92, 0.97]	**<.001**	0.94	[0.91, 0.97]	**<.001**
	SCAT3+Biomarkers	0.97	[0.94, 0.99]	**<.001**	0.96	[0.93, 0.99]	**<.001**
24-48 Hours Post-injury	GFAP	0.57	[0.51, 0.63]	**.033**	0.57	[0.50, 0.64]	**.047**
	UCH-L1	0.51	[0.44, 0.59]	.709	0.51	[0.44, 0.59]	.756
	NF-L	0.49	[0.43, 0.56]	.81	0.47	[0.40, 0.54]	.371
	tau	0.36	[0.30, 0.43]	**<.001**	0.40	[0.33, 0.47]	**.006**
	GFAP+UCH-L1	0.55	[0.48, 0.62]	.165	0.58	[0.50, 0.65]	.057
	All Biomarkers	0.65	[0.57, 0.72]	**<.001**	0.65	[0.57, 0.72]	**<.001**
	SAC	0.60	[0.54, 0.66]	**.001**	0.58	[0.52, 0.65]	**.019**
	BESS	0.67	[0.61, 0.73]	**<.001**	0.61	[0.54, 0.67]	**.002**
	SCAT3	0.91	[0.88, 0.95]	**<.001**	0.93	[0.90, 0.96]	**<.001**
	SCAT3+Biomarkers	0.91	[0.88, 0.95]	**<.001**	0.93	[0.90, 0.96]	**<.001**

GFAP: glial fibrillary acidic protein; UCH-L1: ubiquitin C-terminal hydrolase-L1; NF-L: neurofilament light; SCAT3=Sport Concussion Assessment Tool – 3rd Edition; SAC: Standardized Assessment of Concussion; BESS: Balance Error Scoring System; BSI: CI: confidence interval; AUC: area under the curve.
[a]*P*-values represent significance of area under the curve (AUC) from the null (AUC = 0.5)

© 2020 McCrea M et al. *JAMA Network Open*.

eTable 8. Biomarker Characteristics for Concussed Athletes With or Without Loss of Consciousness or Post-Traumatic Amnesia at All Time Points

		LOC/PTA+					LOC/PTA-				
		n	Mean	SD	Median	IQR [Q1-Q3]	n	Mean	SD	Median	IQR
Baseline	GFAP	54	64.44	23.22	59.54	[45.04, 80.33]	189	61.06	25.42	56.53	[43.92, 72.81]
	UCH-L1	47	12.20	10.81	9.03	[4.11, 15.29]	147	21.02	28.81	11.68	[5.42, 29.09]
	NF-L	54	6.56	2.95	5.67	[4.68, 7.83]	189	6.56	3.42	5.89	[4.29, 7.76]
	tau	54	0.94	0.52	0.86	[0.60, 1.13]	186	1.07	0.72	0.88	[0.61, 1.28]
Acute Post-injury	GFAP	26	215.20	197.40	122.05	[71.71, 311.21]	74	91.92	69.85	71.03	[52.39, 105.49]
	UCH-L1	25	34.45	25.06	31.22	[11.36, 57.07]	67	25.10	22.88	18.81	[8.26, 34.17]
	NF-L	26	8.25	4.24	6.52	[5.41, 9.55]	74	6.61	2.82	6.08	[4.56, 7.85]
	tau	26	1.54	1.31	1.32	[0.68, 1.76]	74	1.27	1.31	0.96	[0.68, 1.53]
24-48 Hours Post-injury	GFAP	37	138.19	178.79	80.97	[58.84, 126.11]	146	91.26	212.67	63.5	[48.84, 91.84]
	UCH-L1	31	17.61	14.33	11.49	[5.90, 30.50]	120	15.37	17.02	9.85	[5.42, 19.39]
	NF-L	37	7.51	3.81	6.13	[5.19, 9.33]	146	6.27	3.02	5.47	[4.34, 7.58]
	tau	37	0.72	0.38	0.64	[0.44, 0.94]	141	0.79	0.68	0.63	[0.39, 0.97]
Asymptomatic	GFAP	53	85.72	55.30	74.34	[57.50, 96.46]	178	68.77	36.12	60.58	[46.48, 82.54]
	UCH-L1	42	13.16	12.41	9.30	[4.19, 17.03]	134	14.83	25.14	9.36	[3.86,17.99]
	NF-L	53	8.82	5.47	7.39	[5.33, 11.03]	178	6.53	6.08	5.73	[4.16, 7.55]
	tau	52	0.93	0.77	0.77	[0.48, 1.06]	175	0.98	0.95	0.75	[0.47, 1.18]
7 Days Post-RTP	GFAP	42	78.35	33.70	67.02	[53.51, 99.31]	159	63.92	21.75	60.37	[47.99, 76.73]
	UCH-L1	33	16.72	19.69	9.99	[4.05, 23.16]	127	15.29	13.15	10.75	[5.58, 23.28]
	NF-L	42	12.98	11.34	8.64	[5.26, 18.20]	159	6.20	2.62	5.80	[4.06, 7.58]
	tau	42	1.13	1.04	0.81	[0.52, 1.24]	158	1.32	2.68	0.93	[0.55, 1.32]

LOC: loss of consciousness; PTA: post-traumatic amnesia; GFAP: glial fibrillary acidic protein; UCH-L1: ubiquitin C-terminal hydrolase-L1; NF-L: neurofilament light; SD: standard deviation; IQR: interquartile range; RTP: return to play

Units represent pg/mL for all biomarkers. Biomarker characteristics for contact and non-contact control athletes are reported in eTable 4.

GFAP: limit of detection (LOD) = 0.221 pg/mL, lower limit of quantification (LLOQ) = 0.467 pg/mL, upper limit of quantification (ULOQ) = 850 pg/mL; UCH-L1: LOD = 1.74 pg/mL, LLOQ = 5.45 pg/mL, ULOQ = 8855 pg/mL; NF-L: LOD = 0.104 pg/mL, LLOQ = 0.241 pg/mL, ULOQ = 429 pg/mL; tau: LOD = 0.024 pg/mL, LLOQ = 0.053 pg/mL, ULOQ = 84.9 pg/mL

© 2020 McCrea M et al. *JAMA Network Open*.

eTable 9. Within-Subjects Comparisons of Biomarkers in Athletes With or Without Loss of Consciousness or Post-Traumatic Amnesia

			LOC/PTA+		LOC/PTA-	
	Time point Comparison		Mean Difference[a]	*P*	Mean Difference	*P*
GFAP	Baseline vs.	Acute Post-Injury	-0.803	**<.001**	-0.302	**<.001**
		24-48 Hours Post-Injury	-0.481	**<.001**	-0.195	**<.001**
		Asymptomatic	-0.212	**<.001**	-0.098	**.003**
		7 Days Post-RTP	-0.120	.242	-0.082	**.032**
	Acute Post-Injury vs.	24-48 Hours Post-Injury	0.322	**<.001**	0.108	**.047**
		Asymptomatic	0.592	**<.001**	0.205	**<.001**
		7 Days Post-RTP	0.684	**<.001**	0.221	**<.001**
	24-48 Hours Post-Injury vs.	Asymptomatic	0.269	**<.001**	0.097	**.009**
		7 Days Post-RTP	0.361	**<.001**	0.113	**.002**
	Asymptomatic vs.	7 Days Post-RTP	0.092	.825	0.016	>.999
UCH-L1	Baseline vs.	Acute Post-Injury	-0.838	**<.001**	-0.309	.082
		24-48 Hours Post-Injury	-0.246	>.999	0.244	.108
		Asymptomatic	-0.058	>.999	0.450	**<.001**
		7 Days Post-RTP	-0.011	>.999	0.121	>.999
	Acute Post-Injury vs.	24-48 Hours Post-Injury	0.592	.054	0.553	**<.001**
		Asymptomatic	0.779	**.001**	0.760	**<.001**
		7 Days Post-RTP	0.827	**.001**	0.430	**.003**
	24-48 Hours Post-Injury vs.	Asymptomatic	0.187	>.999	0.206	.339
		7 Days Post-RTP	0.235	>.999	-0.123	>.999
	Asymptomatic vs.	7 Days Post-RTP	0.048	**>.999**	-0.329	**.006**
NF-L	Baseline vs.	Acute Post-Injury	-0.171	.199	-0.039	>.999
		24-48 Hours Post-Injury	-0.102	>.999	0.006	>.999
		Asymptomatic	-0.195	**.006**	0.032	>.999
		7 Days Post-RTP	-0.435	**<.001**	0.001	>.999
	Acute Post-Injury vs.	24-48 Hours Post-Injury	0.069	>.999	0.045	>.999
		Asymptomatic	-0.024	>.999	0.071	.963
		7 Days Post-RTP	-0.264	**.006**	0.039	>.999
	24-48 Hours Post-Injury vs.	Asymptomatic	-0.093	>.999	0.026	>.999
		7 Days Post-RTP	-0.333	**<.001**	-0.005	>.999
	Asymptomatic vs.	7 Days Post-RTP	-0.240	**.001**	-0.032	>.999
tau	Baseline vs.	Acute Post-Injury	-0.345	.054	-0.179	.128
		24-48 Hours Post-Injury	0.272	.122	0.334	**<.001**
		Asymptomatic	0.160	.986	0.190	**.004**
		7 Days Post-RTP	-0.045	>.999	-0.008	>.999
	Acute Post-Injury vs.	24-48 Hours Post-Injury	0.618	**<.001**	0.513	**<.001**
		Asymptomatic	0.506	**<.001**	0.370	**<.001**
		7 Days Post-RTP	0.301	.211	0.171	.206
	24-48 Hours Post-Injury vs.	Asymptomatic	-0.112	>.999	-0.143	.138
		7 Days Post-RTP	-0.317	.067	-0.342	**<.001**
	Asymptomatic vs.	7 Days Post-RTP	-0.205	.504	-0.199	**.004**

[a]Mean differences of natural log-transformed biomarker values.
Results are reported only for comparisons with significant simple main effects. All *P*-values are corrected for multiple comparisons (Bonferroni).

© 2020 McCrea M et al. *JAMA Network Open*.

eTable 10. Between-Subjects Comparisons of Biomarkers in Athletes With or Without Loss of Consciousness or Post-Traumatic Amnesia, Contact Control, and Non-Contact Control Athletes

	Group Comparison		Acute Post-Injury		24-48 Hours Post-Injury		Asymptomatic		7 Days Post-RTP	
			Mean Difference[a]	*P*	Mean Difference	*P*	Mean Difference	*P*	Mean Difference	*P*
GFAP	LOC/PTA+ vs.	LOC/PTA-	0.583	**<.001**	0.368	**<.001**	0.196	**.018**	NS	
		Contact Control	0.857	**<.001**	0.482	**<.001**	0.253	**.002**		
		Non-Contact Control	0.816	**<.001**	0.468	**<.001**	0.193	**.048**		
	LOC/PTA- vs.	Contact Control	0.274	**<.001**	0.113	.134	0.057	>.999		
		Non-Contact Control	0.233	**.001**	0.099	.405	-0.003	>.999		
	Contact Control vs.	Non-Contact Control	-0.041	>.999	-0.014	>.999	-0.060	>.999		
UCH-L1	LOC/PTA+ vs.	LOC/PTA-	0.207	>.999	NS		0.188	>.999	NS	
		Contact Control	0.725	**.005**			-0.061	>.999		
		Non-Contact Control	0.611	**.044**			-0.234	>.999		
	LOC/PTA- vs.	Contact Control	0.518	**.005**			-0.249	.418		
		Non-Contact Control	0.404	.094			-0.422	**.027**		
	Contact Control vs.	Non-Contact Control	-0.114	>.999			-0.173	>.999		
NF-L	LOC/PTA+ vs.	LOC/PTA-	NS		NS		0.290	**<.001**	0.498	**<.001**
		Contact Control					0.248	**.007**	0.481	**<.001**
		Non-Contact Control					0.200	.075	0.448	**<.001**
	LOC/PTA- vs.	Contact Control					-0.042	>.999	-0.017	>.999
		Non-Contact Control					-0.090	.748	-0.050	>.999
	Contact Control vs.	Non-Contact Control					-0.048	>.999	-0.034	>.999
tau	LOC/PTA+ vs.	LOC/PTA-	0.089	>.999	-0.016	>.999	NS		NS	
		Contact Control	0.294	.200	-0.297	.104				
		Non-Contact Control	0.330	.128	-0.230	.460				
	LOC/PTA- vs.	Contact Control	0.206	.179	-0.281	**.005**				
		Non-Contact Control	0.241	.107	-0.214	.112				
	Contact Control vs.	Non-Contact Control	0.036	>.999	0.067	>.999				

[a]Mean differences of natural log-transformed biomarker values.
NS, Non-significant simple main effect.
Results are reported only for comparisons with significant simple main effects. All *P*-values are corrected for multiple comparisons (Bonferroni).

© 2020 McCrea M et al. *JAMA Network Open*.

eTable 11. Subgroup Area Under the Curve (AUC) for Biomarkers SAC and BESS at Acute Post-Injury and 24-48 Hours Post-Injury Time Points

		LOC/PTA+ vs. LOC/PTA-			LOC/PTA+ vs. Contact Control			LOC/PTA vs. Non-Contact Control		
		AUC	95% CI	*P*[a]	AUC	95% CI	*P*	AUC	95% CI	*P*
Acute Post-injury	GFAP	0.71	[0.59, 0.83]	**.002**	0.81	[0.71, 0.91]	**<.001**	0.81	[0.71, 0.92]	**<.001**
	UCH-L1	0.63	[0.50, 0.76]	.062	0.74	[0.63, 0.85]	**<.001**	0.73	[0.61, 0.85]	**.001**
	NF-L	0.60	[0.48, 0.73]	.120	0.64	[0.53, 0.74]	.029	0.61	[0.49, 0.73]	.083
	tau	0.57	[0.44, 0.71]	.264	0.59	[0.47, 0.72]	.132	0.64	[0.51, 0.77]	**.030**
	GFAP+UCH-L1	0.73	[0.61, 0.85]	**.001**	0.84	[0.74, 0.94]	**<.001**	0.84	[0.73, 0.94]	**<.001**
	All Biomarkers	0.73	[0.61, 0.85]	**.001**	0.85	[0.75, 0.94]	**<.001**	0.83	[0.73, 0.94]	**<.001**
	SAC	0.65	[0.55, 0.75]	**.003**	0.75	[0.66, 0.84]	**<.001**	0.75	[0.65, 0.84]	**<.001**
	BESS	0.53	[0.42, 0.63]	.653	0.69	[0.59, 0.79]	**<.001**	0.66	[0.55, 0.76]	**.005**
	SCAT3	0.54	[0.45, 0.64]	.395	0.94	[0.90-0.99]	**<.001**	0.94	[0.89, 0.99]	**<.001**
24-48 Hours Post-injury	GFAP	0.66	[0.56, 0.76]	**.003**	0.70	[0.61, 0.80]	**<.001**	0.70	[0.60, 0.80]	**<.001**
	UCH-L1	0.56	[0.44, 0.68]	.296	0.56	[0.44, 0.68]	.285	0.57	[0.44, 0.70]	.275
	NF-L	0.60	[0.49, 0.70]	.065	0.57	[0.47, 0.68]	.175	0.55	[0.44, 0.66]	.351
	tau	0.52	[0.42, 0.62]	.704	0.36	[0.26, 0.45]	**.007**	0.42	[0.30, 0.51]	.089
	GFAP+UCH-L1	0.66	[0.55, 0.77]	**.006**	0.69	[0.57, 0.80]	**.002**	0.71	[0.60, 0.82]	**.001**
	All Biomarkers	0.66	[0.56, 0.77]	**.006**	0.70	[0.59, 0.81]	**.001**	0.72	[0.60, 0.83]	**<.001**
	SAC	0.48	[0.38, 0.59]	.739	0.58	[0.48, 0.69]	.114	0.56	[0.45, 0.67]	.240
	BESS	0.55	[0.46, 0.65]	.273	0.71	[0.62, 0.80]	**<.001**	0.65	[0.55, 0.75]	**.005**
	SCAT3	0.50	[0.41, 0.59]	.969	0.92	[0.88, 0.99]	**<.001**	0.95	[0.90, 1.00]	**<.001**

LOC: loss of consciousness; PTA: posttraumatic amnesia; GFAP: glial fibrillary acidic protein; UCH-L1: ubiquitin C-terminal hydrolase-L1; NF-L: neurofilament light; SCAT3=Sport Concussion Assessment Tool – 3rd Edition; SAC: Standardized Assessment of Concussion; BESS: Balance Error Scoring System; BSI: CI: confidence interval; AUC: area under the curve.

[a]*P*-values represent significance of area under the curve (AUC) from the null (AUC = 0.5)

© 2020 McCrea M et al. *JAMA Network Open*.
